# Editorial: Neurodevelopmental, neuropsychiatric and psychosocial correlates of joint hypermobility and related disorders

**DOI:** 10.3389/fpsyt.2022.1109515

**Published:** 2022-12-12

**Authors:** Vincent Guinchat, Carolina Baeza-Velasco, Antonio Bulbena, Marco Castori

**Affiliations:** ^1^Psychiatric Section of Mental Development, Psychiatric University Clinic, Lausanne University Hospital, Lausanne, Switzerland; ^2^Laboratoire de Psychopathologie et Processus de Santé, Université Paris Cité, Boulogne-Billancourt, France; ^3^Department of Emergency Psychiatry and Acute Care, CHU Montpellier, Montpellier, France; ^4^Institute of Functional Genomics, University of Montpellier, CNRS, INSERM, Montpellier, France; ^5^Department of Psychiatry and Forensic Medicine, Universitat Autonoma de Barcelona, Barcelona, Spain; ^6^Anxiety Unit, Hospital del Mar, Institute Neuropsychiatry and Addictions, Barcelona, Spain; ^7^Division of Medical Genetics, Fondazione IRCCS-Casa Sollievo della Sofferenza, Foggia, Italy

**Keywords:** joint hypermobility, Ehlers-Danlos syndromes, neurodevelopmental disorders, psychopathology, psychology

This Research Topic is concerned with the intriguing overlap between apparently separated aspects of human variability: congenital contortionism (a.k.a. generalized joint hypermobility; GJH) on one hand, and neurodevelopmental disorders (NDD) and neuropsychiatric conditions (NC) on the other hand. Literature in this area is still sparse, and we are excited to gather new empirical data. As treated by two separate fields of Medicine, the concurrence of these conditions run under recognized for a long time. Accordingly, individuals seemingly manifesting these features (i.e., GJH plus NDD and/or GJH plus NC) are difficult to address to adequate services and likely to be excluded from the mainstream research. GJH, NDD, and some NC (e.g., anxiety disorders, and depression) are frequent in the general population. So, why an association between GJH and NDD, or GJH and NC should be worth of attention? Is their concurrence in the same individual a casual association or rather the effect of some pre-existing etiopathogenetic relationship? At this point, we can conceive three cooperating explanations:

- Firstly, while GJH is often a harmless trait, it may predict the development of a range of musculoskeletal and neuromotor manifestations including bodily pain, motor delay, coordination troubles and fatigue (i.e., symptomatic GJH). Therefore, one could hypothesize that the neurodevelopmental and neuropsychiatric co-morbidities are the cognitive and behavioral correlates of the physical complaints, which in turn are directly related to the congenital nature of GJH.
- Secondly, GJH, NDD, and NC, involve a physical comorbid burden, and share some frequent associated disorders (e.g., immune dysregulation, dysautonomia, and irritable bowel syndrome). Some of these disorders may not only mediate the relationship between GJH, NDD, and NC, but also can trigger, mimic, or aggravate psychiatric conditions such as depression and ultimately worsen physical disability. In this perspective, GJH should be considered as a warning sign to explore more carefully the presence of impairing physical symptoms in NDD and NC.
- Thirdly, practice and scientific evidence support a high hereditary burden for GJH, NDD, and some NC. Therefore, we can assume that, at least in specific circumstances, the concurrence of GJH, NDD/NC is the result of pleiotropy, which defines the capability that a single gene has to affect the development and functions of different tissues and organs at the same time. In this scenario, we recognize that an increasing number of genes that have been and will be associated with Mendelian variants of NDD as a result of a primary defect of developing brain, equally affect the mesodermal derivatives contributing to joint formation and, hence, cause GJH.

Both symptomatic GJH and NDD show some continuity with normal variability. Mendelian hereditary connective tissue disorders, such as the rare variants of Ehlers-Danlos syndromes (EDS; i.e., all subtypes except for hypermobile EDS—hEDS), are currently considered the most common monogenic disorders featuring GJH (1). This could generate the false impression that rare EDS subtypes are the most common diagnoses among individuals with symptomatic GJH. Currently, most individuals who are referred to specialized clinics for symptomatic GJH are not affected by a monogenic hereditary connective tissue disorder. They usually present a specific phenotypes of GJH with one or more associated musculoskeletal and/or neuromotor manifestations, but they do not have any causative mutation in the disease-genes currently linked to monogenic hereditary connective tissue disorders. On the contrary, they are affected by hEDS, which is an attenuated syndromic presentation resembling the other rarer EDS variants and ascertained by the application of the criteria published in the 2017 International Classification of EDS and related disorders (2). Nevertheless, presumably no <50% of people with a specific phenotypes of symptomatic GJH do not address the criteria for hEDS. For these individuals the background diagnosis are the hypermobility spectrum disorders (HSD) (3). Asymptomatic GJH, HSD, and hEDS are currently conceived as expressions of a unique spectrum of phenotypes. Accordingly, HSD and hEDS are “spectrum disorders,” while asymptomatic GJH represents the harmless extreme of such a spectrum, which, in turn, is intermingled with normal variability (3). Individuals with HSD and hEDS often present a range of associated non-musculoskeletal and neuromotor attributes which include and are not limited to cardiovascular dysautonomia, gastrointestinal functional disorders, bladder dysfunction, and psychiatric co-morbidities (4). Among the most studied psychiatric co-morbidities there are anxiety and depression (5). More recently, an association with eating disorders was put forward (6). In addition, as GJH is a congenital trait, some preliminary evidence suggests a relationship with simple motor delay, speech disorders (7), developmental coordination disorders (8, 9), and attention deficit/hyperactivity disorders (10).

In this regard, the body-mind interconnections observed in the context of GJH, HSD, and hEDS and supported by several studies [e.g., (11, 12)], are illustrated to some extend in the model called “Neuroconnective endophenotype” (13). This model integrates the somatic and psychological characteristics presented by people with spectrum disorders (i.e., GJH without musculoskeletal complaints, HSD, and hEDS) and anxiety, by organizing them in five dimensions: (1) sensorial sensitivity (e.g., altered sensitivity concerning body awareness components such as interoception), (2) body sign and symptoms such as dislocations, easy bruising, and dysautonomia. (3) Somatic conditions including irritable bowel syndrome, chronic fatigue, fibromyalgia among others. (4) Polar behavioral strategies (e.g., supercontrol-uncontrol and avoidance-intrusion), and (5) psychological and psychopathological dimensions (e.g., anxiety and depression). This theoretical proposal highlights the need to evaluate patients with HSD and hEDS who are seen in rheumatological and medical genetics settings as well as individuals primarily assessed by the psychiatrist/clinical psychologist from an integrated perspective (somatic and psychological).

We hope that the following articles, which compose this Research Topic, will contribute to add elements to the disentanglements of these complex clinical situations, and reduce the delays for their recognition and management.

Ishiguro et al.'s paper will first offer an updated critique of the current data available demonstrating an association in various NND and NC (autism, attention deficit/hyperactivity disorder, anorexia nervosa, addiction, anxiety disorders, mood disorders, and personality disorders) in people with spectrum disorders. Whether these symptoms are secondary to the psychological burden of chronic pain, fatigue, physical restriction, aesthetic stigma, or adverse life experience due to GJH (parental overprotection, bullying experience, and social incomprehension), remain an open question but the therapeutic implications are discussed.

Lamari et al. assess the psychosocial correlates of the functional limitations of movements related to the severity of joint hypermobility. Authors used data obtained through analyses of descriptive and inferential crossings of 482 medical records for an observational, quantitative, and cross-sectional study. They show GJH consequences on body mechanics and motor performance in daily, instrumental, recreational, sports, and occupational activities leading to social stigmata. They also corroborate the impact of pain and fatigue since childhood, as unrecognized features of JH leading to suboptimal management.

By screening 53 adults with Eating Disorders (ED; mostly with anorexia nervosa), Baeza-Velasco et al. show that 41% are positive for GJH (comparing with 20% in the general population). Moreover, the comparison between ED + GJH patients and ED only patients indicated a clinical ED subtype that differ with respect to cognitive style: less cognitive rigidity or control and restricting behaviors in the group ED + GJH. This may mirror a distinct causal pathway leading to eating symptoms probably mediated by gastrointestinal problems (e.g., cramps, trouble swallowing, and food allergies) and sensorial anomalies that may impact the body image. In this group “ED is probably secondary to the connective tissue problems rather than attributable to the premorbid perfectionism and rigid cognitive style classically described and requires a specific management.”

Csecs et al. followed a “Neurodivergent approach,” that admits a frequent diagnostic overlap in NDD. They postulated a transnosographic effect of symptomatic GJH on development and tested for increased prevalence of joint hypermobility, autonomic dysfunction, and musculoskeletal symptoms in 109 adults with neurodevelopmental condition diagnoses and a comparison group. Odds ratio for presence of hypermobility in neurodivergent group, compared to the general population was 4.51 (95% CI: 2.17–9.37), more symptoms of orthostatic intolerance and musculoskeletal skeletal pain than the comparison group. Moreover, the GJH severity mediate the relationship between neurodivergence and symptoms of both dysautonomia and pain.

Alternatively, Bejerot et al. in a case-control study including 105 participants, found no differences in GJH between three groups: a group of children with pediatric acute-onset neuropsychiatric syndrome (an umbrella term describing acute psychiatric episodes probably linked to inflammation and associated with postural orthostatic tachycardia syndrome), individuals with any mental disorders, and healthy controls. These preliminary results tend to guide future studies toward the subgroup of patients with developmental traits, rather than acute disorder.

Finally, Glans et al. explored in more details the association between GJH and autism in 199 individuals with autism spectrum disorders (ASD) and 419 non-ASD community controls, assessed for HSD, ADHD, and physical comorbid symptoms. They confirm an association between ASD and GJH that seems primarily driven by a comorbid ADHD, whereas a stronger association is between ASD and symptomatic GJH.

All these papers add elements on our understanding of the far-reaching manifestations of GJH on human normal and abnormal variability. The authors focused of the wide range of possible associations in the fields of neurodevelopment, cognition and behavior. The study of the psychiatric and psychological relationships of GJH seems to offer a privileged scenario for exploring the body-mind connections that exist at the basis of human development and disease. The authors pointed the necessity of future well-designed researches to more deeply explore the etiology, pathogenesis and management of psychiatric, cognitive and behavior manifestations in individuals with GJH.

In conclusion, the identification of GJH in people with NDD and some NC may represent a clue for suspecting a wider phenotype with intricate pathophysiological intersections between neurodevelopment and body habitus. In this category of people usually referred for a previous diagnosis or a suspicion of NDD, prompt identification of GJH might help in early diagnosing concurrent musculoskeletal manifestations which may contribute to, or worsen the leading medical issue. The findings presented in these papers, together with the previous literature, are opening the path to explore the connections between GJH, the neurodevelopment and psychopathology with the final aim to offer a tailored and, hence, more effective treatments to our patients.

## Author contributions

All authors listed have made a substantial, direct, and intellectual contribution to the work and approved it for publication.
